# Advances and Challenges in Heavy-Metal-Free InP Quantum Dot Light-Emitting Diodes

**DOI:** 10.3390/mi13050709

**Published:** 2022-04-30

**Authors:** Xiaojie Jiang, Zhen Fan, Li Luo, Lishuang Wang

**Affiliations:** 1 School of Resources, Environment and Materials, Guangxi University, Nanning 530004, China; jiangxiaojie2321@163.com (X.J.); a912052049@163.com (Z.F.) ; luoli12021@163.com (L.L.); 2 Guangxi Key Lab of Processing for Nonferrous Metals and Featured Materials and Key Lab of New Processing Technology for Nonferrous Metals and Materials, Nanning 530004, China

**Keywords:** colloidal quantum dots, QLED, InP quantum dots, heavy-metal-free materials, InP

## Abstract

Light-emitting diodes based on colloidal quantum dots (QLEDs) show a good prospect in commercial application due to their narrow spectral linewidths, wide color range, excellent luminance efficiency, and long operating lifetime. However, the toxicity of heavy-metal elements, such as Cd-based QLEDs or Pb-based perovskite QLEDs, with excellent performance, will inevitably pose a serious threat to people’s health and the environment. Among heavy-metal-free materials, InP quantum dots (QDs) have been paid special attention, because of their wide emission, which can, in principle, be tuned throughout the whole visible and near-infrared range by changing their size, and InP QDs are generally regarded as one of the most promising materials for heavy-metal-free QLEDs for the next generation displays and solid-state lighting. In this review, the great progress of QLEDs, based on the fundamental structure and photophysical properties of InP QDs, is illustrated systematically. In addition, the remarkable achievements of QLEDs, based on their modification of materials, such as ligands exchange of InP QDs, and the optimization of the charge transport layer, are summarized. Finally, an outlook is shown about the challenge faced by QLED, as well as possible pathway to enhancing the device performance. This review provides an overview of the recent developments of InP QLED applications and outlines the challenges for achieving the high-performance devices.

## 1. Introduction

Colloidal quantum dots (QDs) are zero-dimensional semiconductor nanocrystals, also known as “artificial atoms”, “superlattices”, and “superatom” [1]. Since the 1990s, researchers have paid attention to the synthesis methods and photoelectric application of QDs. Due to the excellent characteristics, such as narrow linewidth, high fluorescent quantum yield, tunable emission color, and wide color range, QDs are the best candidate for next-generation solid-state light and display field. The conventional QDs include II-VI group (ZnS [2], ZnSe [3], CdS [4], CdSe [5]), III-V group (InP [6], GaAs [7], and InAs [8]), IV group (carbon dots [9] and silicon dots [10]), and perovskite QDs (CH3NH3PbBr3 [11]). Most of them are spherical or quasi-spherical, with a diameter between 2 and 20 nm. Due to the quantum confinement effect, the emission wavelength can be turned by the size or morphology of QDs [1,12,13]. Up until now, the Cd-based QLEDs have shown the best performance of the colloidal quantum dot light-emitting diodes (QLEDs) based on colloidal QDs. The brightness of the red, green, and blue (RGB) Cd-based QLEDs reaches 356,000, 614,000, and 62,600 cd/m^2^ [5], and the external quantum efficiency (EQE) reaches 30.90% [14], 23.90% [15], and 19.80% [16], respectively, which is comparable to the mature organic light-emitting diodes (OLEDs). At a luminance of 100 cd/m^2^, the operational lifetime of RGB Cd-based QLEDs is 2,260,000 h [17], 1,700,000 h [5], and 10,000 h [18], respectively, which is basically suitable for low-brightness displays. However, people pay more and more attention to the biotoxicity of cadmium, which threatens human health and the environment. Therefore, the development of nontoxic and environmentally friendly QD materials has become one of the frontier research hotspots.

InP is a representative direct bandgap semiconductor material. Compared with Cd-based QDs, InP QDs have superior characteristics, such as a larger exciton Bohr radius (stronger quantum confinement effect), wide spectral tunability (from deep blue to near infrared), and larger intrinsic absorption coefficient [19,20]. InP QDs have become an ideal candidate for replacing heavy-metal Cd-based QDs [21,22]. With systematic investigation of mechanism and device physics and the rapid development of materials and device fabrication technologies, the electroluminescence (EL) efficiency of red InP QLEDs has been greatly improved and is comparable to that of Cd-based QLEDs [6,23,24,25]. In 2019, Jang et al. achieved a red InP device with an external quantum efficiency (EQE) of 21.4% and an operational lifetime over 100,000 h [6]. In 2021, Chou et al. reported a green InP device with 16.3% EQE [24]. The EQE of blue InP QLEDs is only 2.8% [26]. InP QLEDs with low toxicity and excellent luminescent properties are generally regarded as one of the most promising devices to replace Cd-based QLEDs. The study of high-performance InP QLEDs is significant to realize the wide color gamut display of environmentally friendly QLEDs [27]. 

Another important EL parameter of QLEDs is the half-height full width (FWHM) of the emission spectrum. The line spectrum of the luminescence can provide quite detailed information about the structure of the luminescent center [28,29]. In the spectral linewidth, each atom contributes to the overall line shape, called heterogeneous linewidths [30]. Due to some physical factors, different resonators have different oscillation frequencies, numbers of atoms, and distribution of emission frequencies, the spectral lines will exhibit corresponding line shapes and widths, which are called inheterogeneous linewidths [30]. In addition, each atom in the system is subjected to time-dependent perturbations (dynamic perturbations) that produce random changes in the amplitude or phase of the resonant oscillator, resulting in a broadening of the spectrum. In addition, dynamic perturbations may also cause random changes in the frequency of the leap, resulting in a dynamically inhomogeneous broadening. In general, the line shape obtained from a typical spectroscopic measurement contains both heterogeneous and inhomogeneous broadening. 

The study of spectral linewidth is useful for understanding the variation in the properties of materials and devices, so the half-height full width of the spectrum is an important parameter for measuring the luminescence properties of materials and devices. To obtain high color purity for the QLEDs, the narrower the half-height full width (FWHM) of the emission spectrum, the better. The current mature Cd-based QLED linewidth can reach 20 nm [31]; the Pb-based perovskite QLED, which has received a lot of attention, can achieve an FWHM of less than 20 nm [32]; and the environmentally friendly InP QLED has a quite narrow FWHM of about 30–40 nm (Table 1), showing good color saturation for use as lighting and display arrays.

Green InP/ZnSeS QLEDs were first reported by Lim et al. [34] in 2011. There are two aspects to improve the performance of InP QLEDs: (1) core/shell structure and surface ligand modification of QDs, which requires in-depth research on the growth mechanism of InP QDs; and (2) optical and electrical optimization of InP QLEDs, which refers to the structural design of QLED devices and modification of charge transport layers, such as HTL and ETL. The following text mainly focusses on the above two aspects.

## 2. Synthesis of InP QDs

### 2.1. Synthetic Method

**Figure 1:**
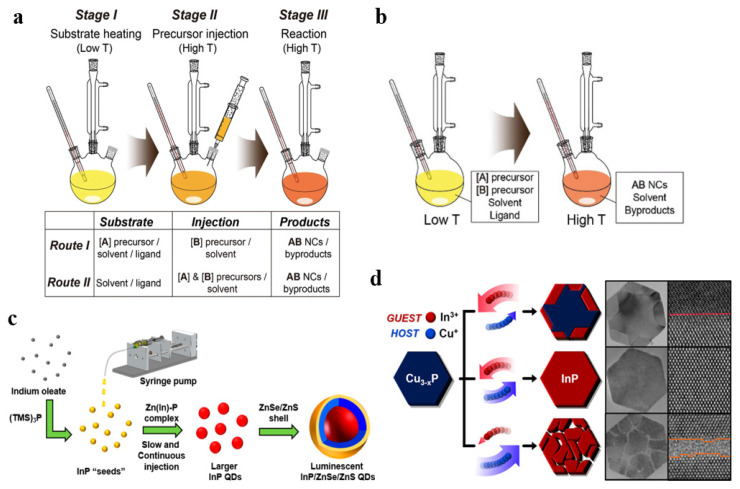
Schematic of QD synthesis by (**a**) hot-injection, (**b**) heat-up (Reprinted with permission from Ref. [46]. © 2020, WILEY-VCH Verlag GmbH & Co. KGaA, Weinheim, Germany), (**c**) seeded growth (Reprinted with permission from Ref. [41]. © 2018, American Chemical Society), and (**d**) cation exchange method (Reprinted with permission from Ref. [44]. © 2019, American Chemical Society).

The synthesis of InP QDs was first reported by Micic et al. in the early 1990s [35]. At first, InP QDs did not receive much attention, although it is not long after the first synthetic reports of Cd-based QDs. In the initial work, InP QDs do not have a shell, and these QDs usually exhibit asymmetric broad emission peaks in photoluminance (PL) spectra, sometimes also characterized by shoulder peaks. One of the challenges in synthesizing high-quality InP nanocrystals is how to control the nanocrystal size distribution by regulating the nucleation and growth processes [36,37]. The current work on InP QDs mostly adopts a direct strategy, in which the In and P precursors are heated and reacted in a solution composed of an organic solvent and a coordination-capped ligand to obtain nanocrystals dispersed in the solvent. Hot-injection and heat-up are two representative methods. A synthetic route diagram of hot-injection method is shown in Figure 1a, which contains either rapidly injecting one [B] precursor into a hot reaction anther [A] precursor medium (Route I) [22], or injecting both [A] and [B] precursors into a hot medium (Route II) [38]. In a supersaturated state, the precursor has a faster nucleation rate. As time proceeds, the concentration of reactants decreases and crystals precipitate. For the heat-up method (Figure 1b), all reactants are dissolved in a solvent, mixed uniformly, and then heated to the reaction temperature [35]. Therefore, using the heat-up method requires strict control of the composition ratio of precursors and ligands, reaction temperature, and heating rate to reduce the overlap of nucleation and time [39].

However, the simultaneous nucleation and crystal growth in the direct synthesis strategy leads to a non-uniform grain size distribution [40]. To solve this problem, the researchers proposed seeded growth strategies [6,41] and the cation exchange method [42,43,44]. The seeded growth method first pre-synthesizes InP QDs seeds and then uses additional In and P precursors for further growth to obtain uniform InP QDs, as shown in Figure 1c. Due to the good controllability of the size, morphology, and uniformity of InP QDs, seed growth is a better choice for obtaining high-quality InP QDs. The cation exchange method (Figure 1d) refers to replacing cations in host nanocrystals with guest ions dissolved in water or organic solvents. For covalent InP QDs, the exchange reaction usually requires thermal stimulation. The low mobility of anions in solution ensures that the size and morphology of the QDs are largely preserved. The cation exchange method is a facile route for the rapid synthesis of size-controllable nanocrystals [45]. However, cation exchange InP QDs usually contain a mass of defects, especially when templated nanocrystals composed of low-valent cations were employed [46]. Representative synthesis methods of InP QDs and EQE of QLEDs are demonstrated in Table 2.

### 2.2. Cost-Effectiveness of InP QDs

Colloidal InP QDs are fabricated by means of wet chemical synthesis. Compared with traditional III–V epitaxial QDs, colloidal InP QDs have obvious advantages, such as a simple synthesis process, low-cost of instrumentation and materials, controllable reaction process, low reaction temperature, solution-process, and large area preparation. These excellent characteristics are the reason why colloidal InP QDs are receiving more and more attention and have become the star materials in the field of wearable devices and lighting displays. However, it is worth noting that, compared with mature colloidal Cd-based QDs, especially perovskite QDs, InP precursor materials are slightly expensive, and the synthesis of InP QDs requires higher reaction safety, so the development of colloidal InP QDs has been limited for quite a long time. Compared with Cd-based and perovskite QDs, whose commercial development is limited by toxicity, InP QDs have always had poor optical properties. The reason is that InP synthesis proceeds in a non-classical synthesis mechanism [50]. In addition, the steeper effective bandgap and nanocrystal size of InP than CdSe, as well as its easy oxidation to form more trap sites, negatively affect its PLQY and FWHM [28,51]. However, effective solutions have been found for the above problems, and they are described in detail below. In recent years, researchers used aminophosphorus precursors to replace phosphorus precursors, which have high reactivity and poor safety. Tris(trimethylsilyl)phosphine ((SiMe3)3P), as the mainstream phosphorus precursor for the synthesis of InP QDs, is not only expensive but also highly active; thus, it can spontaneously ignite in air and explode at high temperatures [35,52]. Recently, a type of aminophosphorus, tris(dimethylamino)phosphine ((DMA)3P), was used to replace (SiMe3)3P as a phosphorus precursor [47,53,54]. By using aminophosphorus precursors, controlled-reaction conditions with suitable reactivity and good safety can be obtained in the wet synthesis process. As a result, the synthesis of indium phosphide has made rapid progress, so that the performances of QLEDs are greatly improved.

## 3. Influence of Core/Shell Structure on Performance of InP QLEDs

**Figure 2:**
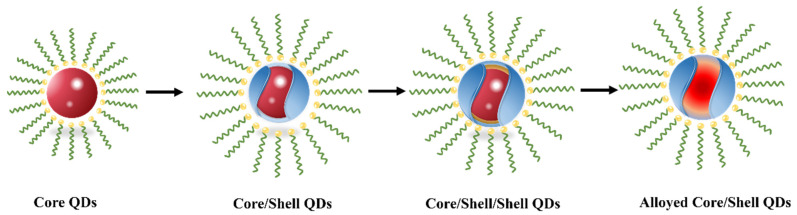
Schematic diagram of the development of InP QDs.

In principle, the emission wavelength of QDs can be regulated by dynamically changing the electronic structure of QDs. The construction of high-quality colloidal QDs is a key factor in determining the EL performance of QLEDs. It is necessary to tailor the InP QD core–shell structure and optimize its device structure to improve the EL performance through nanoengineering. The nanoengineering of InP QDs, that is, the tuning of parameters, such as emission wavelength, linewidth and luminous efficiency of QDs through energy-band engineering of core–shell structures and alloys out-shell. The development of InP QDs can be divided into several stages: from a core-only QD, to a core/shell QD, and then to a more refined core/shell/shell QD and alloyed core/shell QD (Figure 2) to improve the photoluminescence quantum yield (PLQY) and stability of QDs, as well as the EL performance of InP QLEDs.

### 3.1. Core-Only InP QDs

In the early works, QDs often fabricated with a single InP core without the outside shell. In 1994, Nozik et al. firstly synthesized InP QDs by using InCl as the indium source and $P{\left({\mathrm{SiMe}}_{3}\right)}_{3}$ as the phosphorus source [35]. In 1996, Guzelian prepared 2–5 nm InP QDs in trioctylphosphine oxide (TOPO) by using InCl and ${\left(\mathrm{TMS}\right)}_{3}P$ [52]. In the same year, Mićić et al. also prepared InP QDs of 2.5–4.5 nm, which exhibited 30% PLQY at 300 K and 60% PLQY at 10 K high QY [55]. However, the abovementioned InP QDs usually exhibit a wide linewidth, asymmetric spectra, and shoulder peaks. The reason is likely related to surface defects, where electron and hole pairs are prone to non-radiative recombination, and PL emission is largely suppressed [35,55,56]. The synthesis process usually takes 3–7 days, and the long time makes the nucleation and growth processes overlap, resulting in uneven particle size distribution. In 2002, Battaglia et al. synthesized InP QDs with symmetrical single-peak emission spectrum by using indium acetate, instead of indium oxalate, and the synthesis time was decreased to 3 h [22].

**Figure 3:**
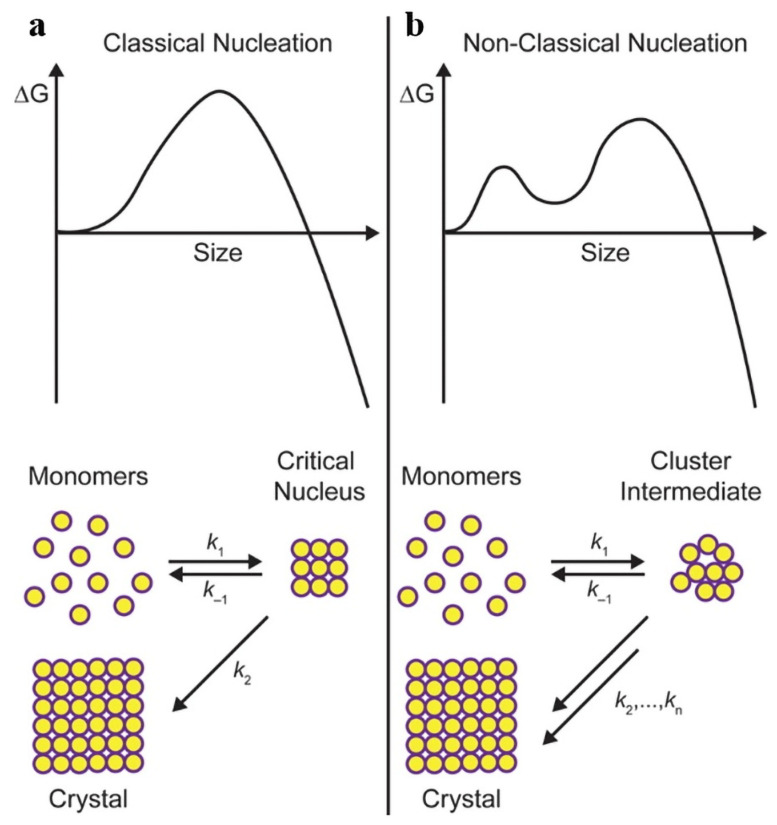
Schematic diagram of free energy as a function of particle size and crystallization for (**a**) classical and (**b**) non-classical nucleation models. Reprinted with permission from Ref. [58]. © 2016, American Chemical Society.

The intermediates InP magic-sized clusters (MSCs) were first observed and reported by Xie et al. in 2009 [57]; they believe that the MSCs with higher thermal stability makes the nucleation and growth process of InP nanocrystals different from the classical model. Cossairt et al. proposes a non-classical view of nucleation growth, as shown in Figure 3 [58]. It is shown that the MSCs play a slow-release role, providing active monomers in reaction, so that the concentration of active monomers can be maintained at a high level [50]. Xie et al. observed the reaction process of InP MSCs and found that the content of MSCs gradually decreased with the increase of reaction temperature and disappeared completely at 300 °C [59]. Using this method, they obtained nearly monodisperse InP QDs. A study on the growth mechanism of InP MSCs will play a positive role in overcoming some challenges of InP QDs, such as monodisperse QDs and precise morphology control. 

### 3.2. Core/Shell Structure

**Figure 4:**
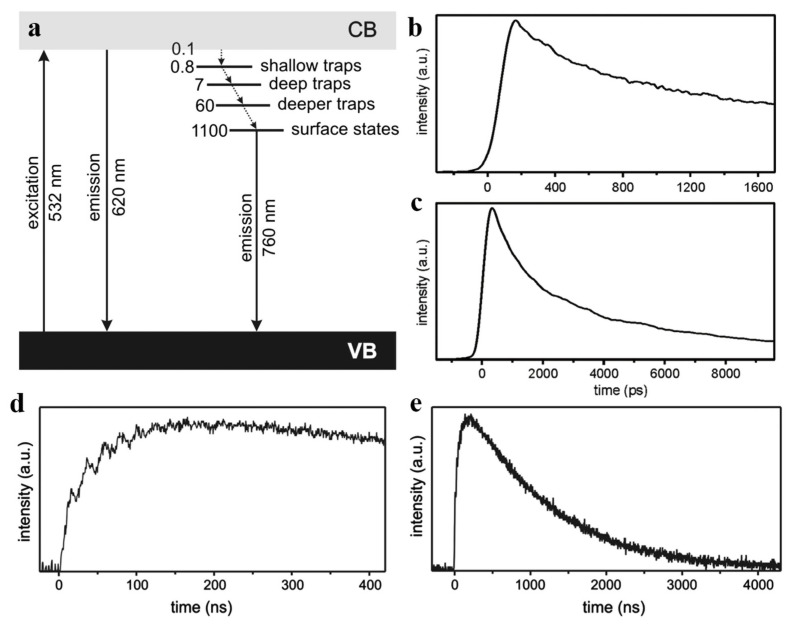
(**a**) Schematic diagram for the luminescence relaxation kinetics of core–shell InP@ZnSe quantum dots dispersed in n-hexane. All the time, constants are given in units of nanoseconds. Picosecond luminescence kinetic profiles were measured in short (**b**) and long time (**c**) windows of core–shell InP@ZnSe nanoparticles dispersed in n-hexane, and the sample was excited at 532 nm and monitored at 600 nm. Nanosecond luminescence kinetic profiles were measured in short (**d**) and long time (**e**) windows of core–shell InP@ZnSe nanoparticles dispersed in n-hexane, and the sample was excited at 532 nm and monitored at 800 nm. Reprinted with permission from Ref. [65]. © 2010, Elsevier Inc.

Due to the poor encapsulation of core-only InP QDs and many superficial defects, the PLQY of InP QDs is quite low. To improve the stability and PLQY of InP QDs, a core/shell heterostructure was fabricated which is covered with an out-shell and organic ligands as surface passivated materials [20,60]. In 2000, Mićić et al. first used perfectly matched ZnCdSe_2_ to the shell [56]. However, the small conduction band minimum (CBM) difference between ZnCdSe_2_ and InP resulted in a weaker electron confinement, and the PLQY was <10%. On the contrary, Haubold et al. used ZnS as the shell, which has a large lattice mismatch but a large band gap (3.68 eV) [61]. Surprisingly, the InP QDs reached a 23% PLQY. It is shown that high-quality shell growth is an effective way to enhance the performance of InP QDs. Under the same strategy, in 2007, Xie et al. developed a low-temperature synthesis method of InP/ZnS QDs, and the PLQY reached 40% [62]. In 2008, a rapid growth method of InP core and ZnS shell was reported, which improved the efficiency of QDs with uniform size [63]. In 2012, Lim et al. synthesized ZnS/InP QDs by etching the InP core with acetic acid [64], and blue InP QDs with an emission wavelength of 475 nm and a FWHM of 39 nm were obtained. Although the performance of InP QDs has been improved, the PLQY and stability are still lower than those of Cd-base QDs. Meanwhile, these reported high-efficiency InP QDs are not used to fabricate InP QLEDs. The investigation of PL kinetic curve reveals the reason (Figure 4) [65]. Due to the large lattice mismatch between the InP core and the ZnS shell, many trap sites are formed. Moreover, most of the electrons are captured by the traps and lost through the non-radiative recombination process.

### 3.3. Core/Shell/Shell Structure

**Figure 5:**
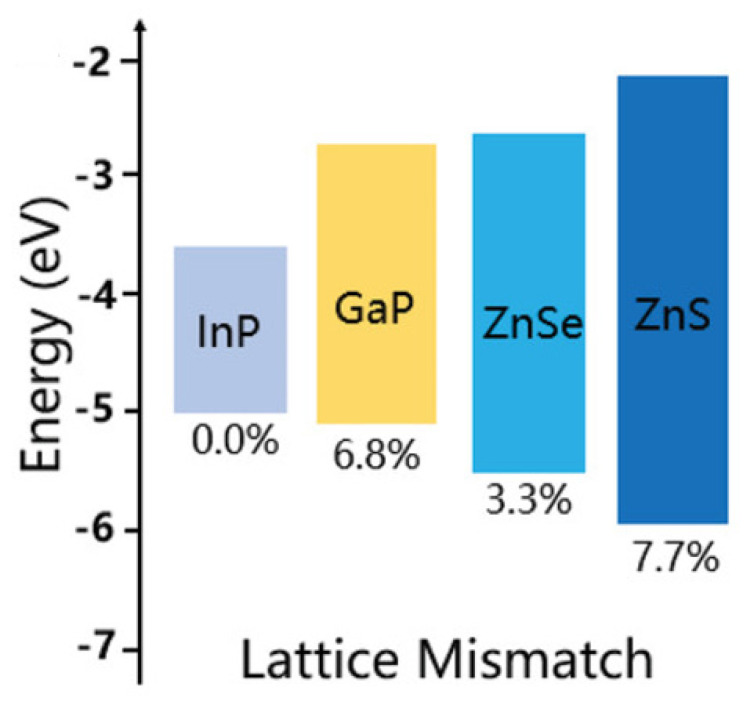
Lattice mismatch between core InP and shell materials of GaP, ZnSe, and ZnS. Reprinted with permission from Ref. [76]. © 2020, Wiley−VCH GmbH.

The reason for the poor performance of InP-based QLEDs is the deep gap state defects and oxidation defects of InP QDs, resulting in low radiative recombination efficiency of electrons and holes injected into the emission layer of InP QDs [20,66,67]. Among them, the oxidation defect of InP is the main reason for the poor optical properties of InP [68]. In addition, the InP/ZnS core/shell structure inevitably leads to the inter-doping of core/shell structure, because the III and V group atoms are easily doped into the II–VI atoms [69,70]. Both oxidation defects and component doping could generate a large number of surface traps at the InP/ZnS interface, as these will cause energy losses, such as Förster resonance energy transfer (FRET) and Auger recombination (AR) [6,71]. To suppress the non-radiative progress, double-shell QDs were fabricated [72]. The conventional shell materials used in InP QDs are GaP, ZnS, ZnSe, and ZnSeS [6,73,74,75,76], as shown in Figure 5. Among them, the lattice mismatch between ZnSe and InP is the smallest, at only 3.3%; GaP reaches 7.1%; and ZnS is the largest, which is 7.7% [70,73,74,77,78]. However, the band gap of ZnS is higher, reaching 3.68 eV [77]. The high band gap attribute InP/ZnS QDs reach a good confinement effect for the electron. Therefore, GaP and ZnSe are regarded as strong competitors for the intermediate shell and ZnS for the out-shell [34,73,77,79]. In 2019, Zhang et al. successfully synthesized InP/GaP/ZnS core/shell/shell QDs which have high stability, a high PLQY (67%), and a large particle size (7.2 ± 1.3 nm) [80].

**Figure 6:**
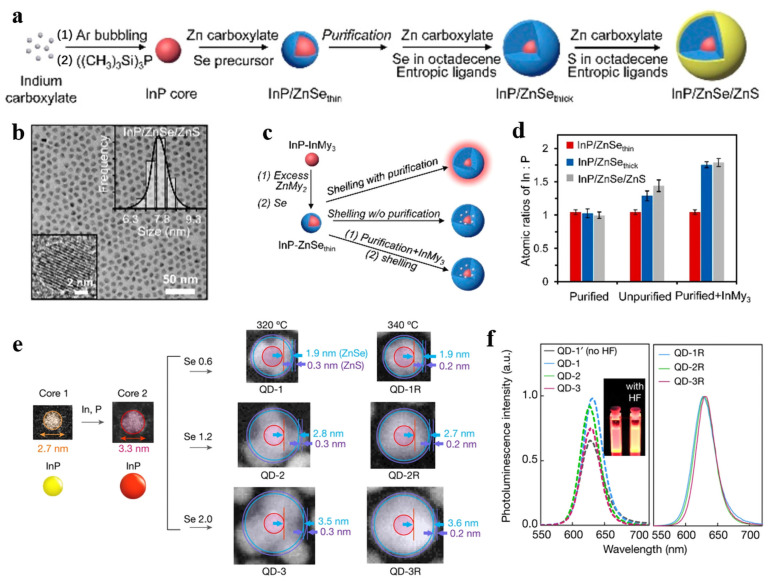
(**a**) Stoichiometry-controlled synthesis scheme. (**b**) TEM images of the obtained InP/ZnSe/ZnS QDs with size-distribution histograms (top right) and high-resolution TEM images (bottom left). (**c**) Stoichiometry-controlled route (top), conventional route (middle), and control-experiment route (bottom) producing different QDs. (**d**) Atomic In:P ratios in InP-based QDs synthesized by three different routes. Reprinted with permission from Ref. [74]. © 2019, American Chemical Society. (**e**) Preparation of InP cores and InP/ZnSe/ZnS QDs with different morphology and shell thickness. (**f**) Photoluminescence spectra of QD-1′ (prepared without HF addition), QD-1, QD-2, QD-3, QD-1R, QD-2R, and QD-3R. Inset, photograph of QD-1′ (no HF) and QD-3 taken under 365 nm illumination. Reprinted with permission from Ref. [6]. © 2019,The Author(s), under exclusive license to Springer Nature Limited.

Double-shell InP/ZnSe/ZnS QDs with a 1 nm ZnS shell were reported in 2011, and the QDs were successfully used as an emitting layer in QLED [34]. In 2016, Lee et al. synthesized a series of trichromatic InP/GaP/ZnS core/shell/shell QDs by using an improved thermal synthesis method [81]. Blue QDs were obtained by using t-DDT to prevent grain growth; the transition from green to orange was obtained by adjusting the content of myristic acid; red QDs were obtained by adding a large amount of gallium chloride. The PLQYs are about 40%, 85%, and 60%, and the FWHMs are 50, 41, and 65 nm for the synthesized blue, green, and red QDs, respectively. The large size and thick shell exhibit a better ability to suppress non-radiative recombination processes. Li et al. confirmed that, In or P doping into the shell also affect the QDs performance, as shown in Figure 6a–d [74]. By controlling the stoichiometry of precursor, red InP QDs show a PLQY over 90% and an FWHM of 35 nm. In addition, the EQE of InP QLEDs reached 12.2%. As demonstrated in Figure 6e,f, in 2019, Won et al. added HF solution to the early stage of shell growth to prevent re-oxidation, and increased the thickness of the ZnSe interlayer to 3.6 nm [6]. Finally, high-quality red InP/ZnSe/ZnS QLEDs based on high spherical QDs were obtained; the highest brightness of the device is 100,000 cd/m^2^, the maximum EQE is 21.4%, and the operational lifetime is 1,000,000 h at 100 cd/m^2^. The EL performance of the InP/ZnSe/ZnS QLEDs is comparable to the state-of-the-art Cd-based QLEDs.

**Figure 7:**
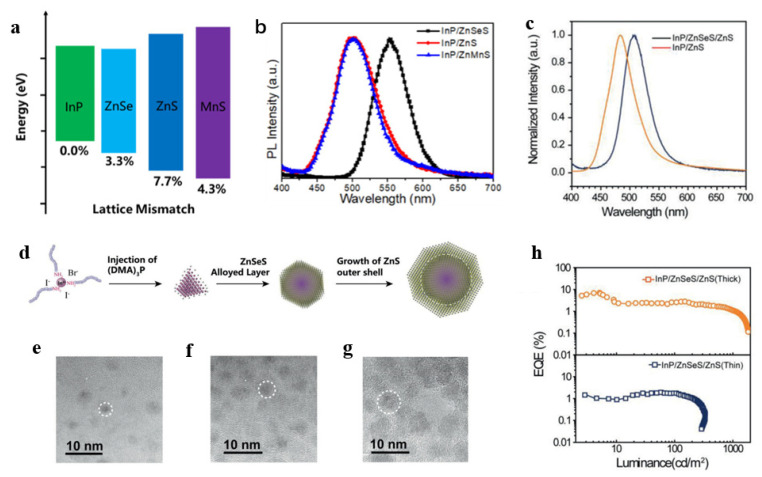
(**a**) Band gap and lattice mismatch of InP, ZnSe, ZnS, and MnS in the prepared green InP QDs. (**b**) PL spectra of InP QDs with different shells. Reprinted with permission from Ref. [83]. © 2019, American Chemical Society. (**c**) PL spectra of InP QDs synthesized with and without ZnSe content. (**d**) The growth stages of green InP QDs synthesized with (DMA)3P. (**e**–**g**) Aliquot study of three growth stages during reactions: InP cores, InP/ZnSeS QDs, and InP/ZnSeS/ZnS QDs. (**h**) EQE versus luminance of the two QLEDs. Reprinted with permission from Ref. [47]. © 2021, Wiley-VCH GmbH.

In 2017, high-performance green InP QLEDs were demonstrated by using thick-shelled InP/ZnSeS/ZnS QDs and a ZnMgO commercial electron transport layer in an inverted structure [82]. The brightness of these improved QLEDs is higher than 10,000 cd/m^2^. Zhang et al. introduced the intermediate ZnMnS layer to obtain green InP/ZnMnS/ZnS QDs with a PLQY of 80% [83]. ZnMnS reduces the lattice mismatch between the core InP and shell ZnS, as shown in Figure 7a,b. In 2019, Zhang et al. synthesized thick-shell InP/GaP/ZnS QDs [80], and the green QLEDs exhibited a maximum current efficiency of 13.7 cd/A and an EQE of 6.3%, which is 1.8 times higher than the previously reported value [84]. In 2021, Liu et al. used ${\left(\mathrm{DMA}\right)}_{3}P$ as the P precursor to synthesize green InP/ZnSeS/ZnS QDs [47] and obtained the highest PLQY of 95%, as shown in Figure 7c–h. Finally, the EQE of the fabricated inverted InP QLEDs exceeds 7%, which is the highest EQE record currently reported based on the ${\left(\mathrm{DMA}\right)}_{3}P$ synthesis route. Recently, Chao et al. synthesized green InP/ZnSe/ZnS QDs with a PLQY of 86% [24]. Then they modified the InP QD emitting layer by passivation with various alkyl diamines and zinc halides, and a record 16.3% EQE of green QLEDs was achieved.

**Figure 8:**
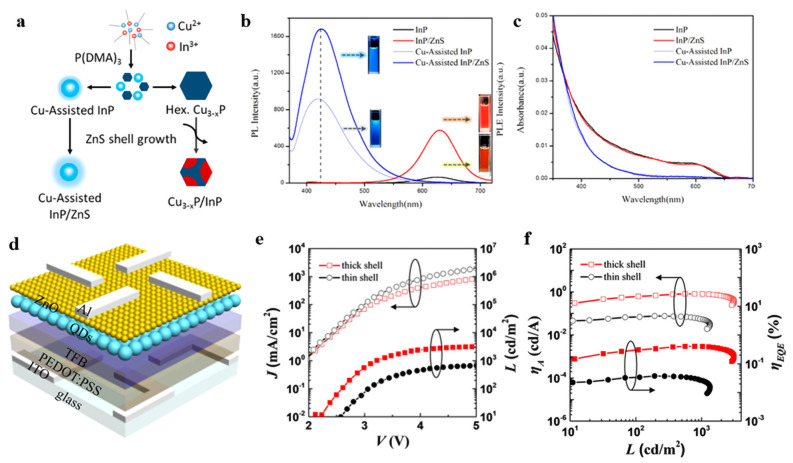
(**a**) Schematic synthesis of blue-emitting InP/ZnS QDs with Cu−assisted process. (**b**) UV−Vis absorption and (**c**) photoluminescence emission spectra of InP and InP/ZnS QDs, Cu−assisted InP QDs, and Cu−assisted InP/ZnS core/shell QDs. Reprinted with permission from Ref. [49]. © 2019, American Chemical Society. (**d**) Schematic diagram of the QLEDs structure. (**e**) Variations of current density and luminance as a function of the voltage. (**f**) Current efficiency and EQE as a function of the luminance. Reprinted with permission from Ref. [85]. © 2020, American Chemical Society.

Compared with green and red InP QDs, the core size of blue InP QDs is smaller, meaning that more defects will be generated. Moreover, it is difficult to realize controllable epitaxial growth of QD shells, resulting in a wide linewidth and low PLQY; the research on high-performance blue InP QDs lags far behind red and green QLEDs. In 2017, Shen et al. used ${\left(\mathrm{DMA}\right)}_{3}P$, which has a moderate reaction rate and is easily tunable as the P precursor, to adjust the size of InP QDs to achieve blue light emission [54]. In addition, the formation of a halamine passivation layer, combined with the zinc halide–mediated colloid method, can greatly reduce surface defects; they obtained InP/ZnS QDs with a PL peak of 477 nm and an FWHM of 43.7 nm. In 2019, Huang et al. found that the copper ions can compete with the nucleation process of InP QDs to form smaller-sized InP QDs. Moreover, deep blue InP QDs were achieved with a record emission wavelength of 425 nm, as shown in Figure 8a–c [49]. In 2020, Zhang et al. used GaP as the intermediate shell to synthesize InP/GaP/ZnS//ZnS core/shell/shell InP QDs with a PLQY of 81%, as shown in Figure 8d–f [85]. The best-performing InP QLED shows an EQE of 1.01%, a maximum luminance of 3120 cd/m^2^, and an FWHM of 50 nm at the luminescence peak at 488 nm. Ding et al. successfully synthesized pure blue InP/ZnS/ZnS core/shell/shell QDs with an emission wavelength of 468 nm, a PLQY of 45%, and an FWHM of 47 nm by using stable and low-cost ${\left(\mathrm{DMA}\right)}_{3}P$, ${\mathrm{ZnI}}_{2}$, and ${\mathrm{InI}}_{3}$ as precursors [76]. The EQE of the device is enhanced from 0.6% of InP/ZnS QLED to 1.7% of InP/ZnS/ZnS QLED.

### 3.4. Alloyed Core/Shell Structure

**Figure 9:**
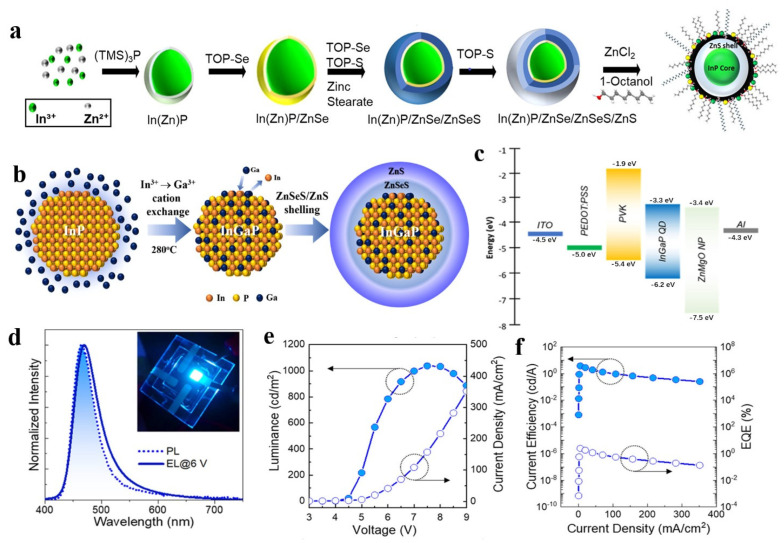
(**a**) Schematic diagram of the two-step heating-up process synthesis of InP QDs. Reprinted with permission from Ref. [87]. © 2021, American Chemical Society. (**b**) Schematic illustration of In^3+^−to−Ga^3+^ cation−exchange−based InGaP core and subsequent ZnSeS/ZnS double shelling. (**c**) Energy band diagram of InGaP QLEDs. (**d**) Spectral comparison of PL of a QD dispersion with EL collected at 6 V. (**e**) Variations of current density and luminance as a function of the voltage. (**f**) Variations of current efficiency and EQE as a function of the current density. Reprinted with permission from Ref. [43]. © 2020, American Chemical Society.

Both oxidation defects and compositional doping will generate many surface traps at the InP/ZnS interface, and this will cause some non-radiative energy losses. Alloyed—especially gradient alloyed QDs—can be effectively used to solve this problem. The lattice mismatch can be gradually changed to alleviate the interface defects that are caused by the lattice strain. The alloyed QDs can effectively inhibit Auger recombination, due to the smooth interface potential distribution. To reduce lattice core–shell mismatch, Pietra et al. realized alloyed In_x_Zn_y_P QDs; the lattice constant can be fine-tuned by changing the Zn^2+^ concentration and reduced from 5.93 to 5.39 Å [86]. Moreover, the maximum PLQY of the core/shell InP QDs with matching lattice parameters reaches 60%. In 2021, Taylor et al. developed a two-step heating-up process to grow an In(Zn)P core and coated inorganic ZnSe/ZnSeS/ZnS shell, as shown in Figure 9a [87]. Surface-modified bright green InP core/shell QDs showed a narrow FWHM of 33 nm and a PLQY of 71%. To surmount intrinsic size limitation, in 2020, Kim et al. implemented action exchange strategy to achieve deep blue InGaP QDs [43]. The 465 nm emitting InGaP/ZnSeS/ZnS QDs were further employed as an emitting layer of an all-solution-processed QLED, and the device generates a maximum EQE of 2.5% and luminance of 1038 cd/m^2^, as shown in Figure 9b–f. By adopting a lowly reactive P precursor, Liu et al. significantly promoted alloyed-shell InP/ZnSeS/ZnS QDs with a PLQY of 95% and an FWHM of 45 nm, which was the record PLQY obtained from the aminophosphine system [47]. By optimizing the device structure, an inverted green InP QLED with an EQE of 7.06% was achieved.

### 3.5. Ligand Engineering

**Figure 10:**
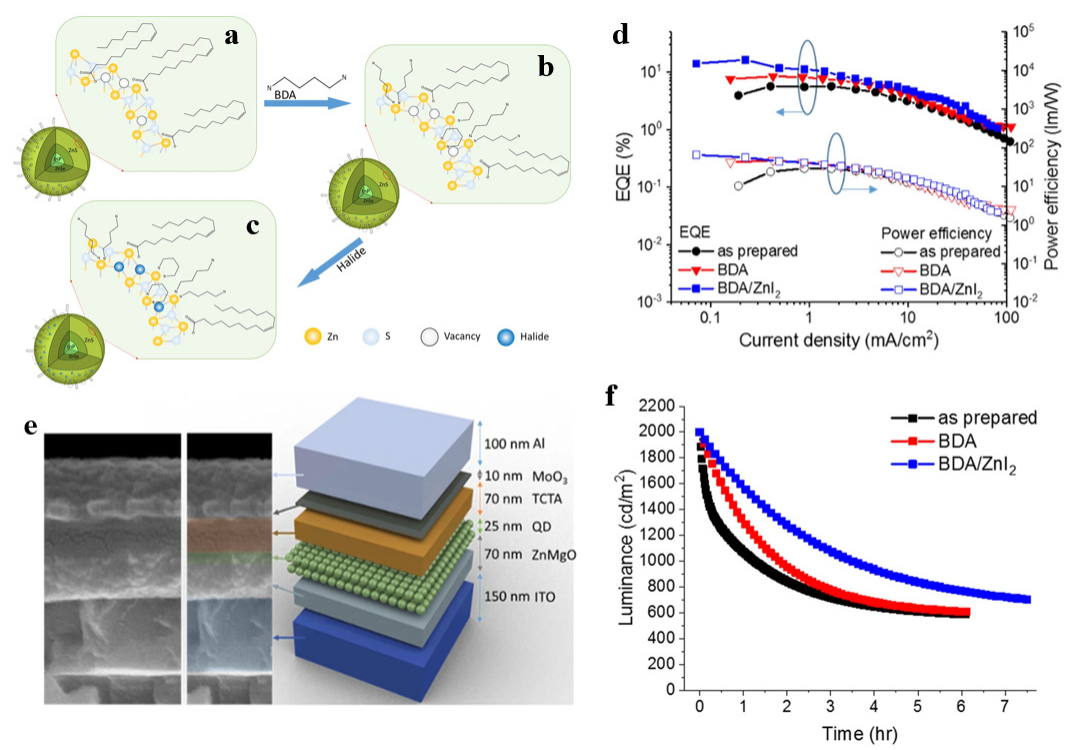
Schematic diagram of the synergistic passivation of InP green QDs by BDA and zinc halide. (**a**) Green InP/ZnSe/ZnS QDs with oleic acid ligands. (**b**) InP QDs modified with BDA consisting of two amino groups. (**c**) InP QDs were further modified by passivation of various zinc halides. (**d**) Variations of EQE and power efficiency as a function of the current density. (**e**) The SEM image of the interlayers cross−section and corresponding device structure. (**f**) Operational lifetimes of InP QLEDs under a condition of 2000 cd/m^2^. Reprinted with permission from Ref. [24]. © 2021, The Author(s).

To improve the photophysical property, surface chemistry modification has long been used to achieve nonblinking and stable QDs. In ligand modification, the ligands affect the superficial defects of QDs, leading to enhanced carrier transport in QD thin films and an improved PLQY [88]. Ligand exchange is one of the key factors affecting carrier mobility; mobility can be modified by decreasing the ligand length [89]. Changing the chemical binding group and dipole moment of the ligand will change the surface dipole strength of the QD ligand, thereby varying the valence band maximum (VBM) and CBM of the QDs [90,91]. Won et al. replaced the oleic acid ligand on the surface of the quantum dots with a shorter chain length hexanoic acid (HA), and the device hole current of the short ligand QDs increases fourfold, promotes exciton recombination, and reduces Auger recombination, and the EQE of the device increases to 21.4% [6]. In 2021, a high EQE of 16.3% for InP QLEDs was obtained based on the modification of surface ligands and the ZnI_2_ precursor, which is the record EQE value of green InP QLEDs up to now [24]. In this work, a shorter chain (BDA) was used to strengthen the molecular bonds between QDs and control carrier transfer at the QDs interface, and hole injection and mobility were modified; the schematic diagram of the reaction is shown in Figure 10. Yoo et al. reported InP QLEDs decorated with bipyridyl ligands with delocalized molecular orbitals, which lowered the charge injection barrier and improved the charge balance in QDs [92]. Recently, a method was reported to encapsulate blue InP QDs with siloxane via 3-Trimethoxysilylpropanethiol (TMSPT) for ligand exchange and condensation reactions to improve their stability [93].

In summary, in order to obtain high-efficiency InP QLEDs, it is necessary to synthesize InP QDs with high lattice integrity, uniform size distribution, and high stability. The development of InP QDs varies from the initial shell-free structure to the core/shell structure that uses a metal shell to passivate the surface of quantum dots, and now to a conventional double-shell structure or alloyed shell structure that can effectively alleviate lattice mutation and reduce core–shell interface defects. In addition, by increasing the thickness of the shell layer, and performing ligand exchange can also improve the efficiency and stability of InP QDs, thereby improving the performance of InP-based QLEDs.

## 4. Influence of Device Structure on the Performance of InP QLEDs

**Figure 11:**
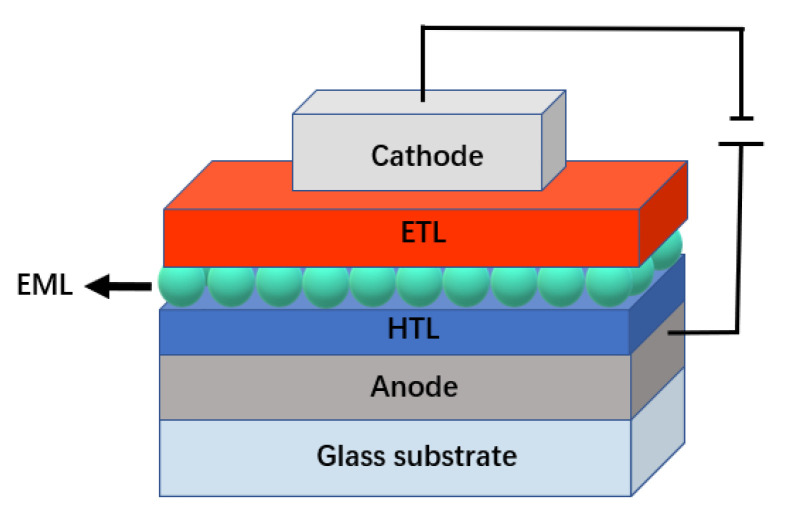
Schematic diagram of QLED structure.

The design and optimization of the device structure determine the injection and transport of carriers and the recombination rate of excitons, as well as the efficiency and brightness of the device. To realize high-efficiency InP QLEDs, the structure of QLEDs needs to be carefully designed, considering a conventional or inverted structure, modification of energy levels of HTL and ETL, interfacial engineering, and the optical optimization. Furthermore, it is also crucial to prevent excessive electron leakage to improve the operating lifetime. QLEDs generally consist of a multilayer sandwich structure: a QD light-emitting layer (EML), an electron transport layer (ETL), a hole transport layer (HTL), and positive and negative electrodes (Figure 11). Operated with the electric field, electrons and holes are respectively transported through the charge transport layer and injected into the QD EML and then formed excitons, which are de-excited by radiative recombination and emit photons. The performance of QLEDs has been greatly improved with the deeply understanding of the device physics and mechanism of QLEDs, the continuous development of QDs and charge transport materials, and the continuous improvement and optimization of device structures.

### 4.1. The Blended Emitting Layer

**Figure 12:**
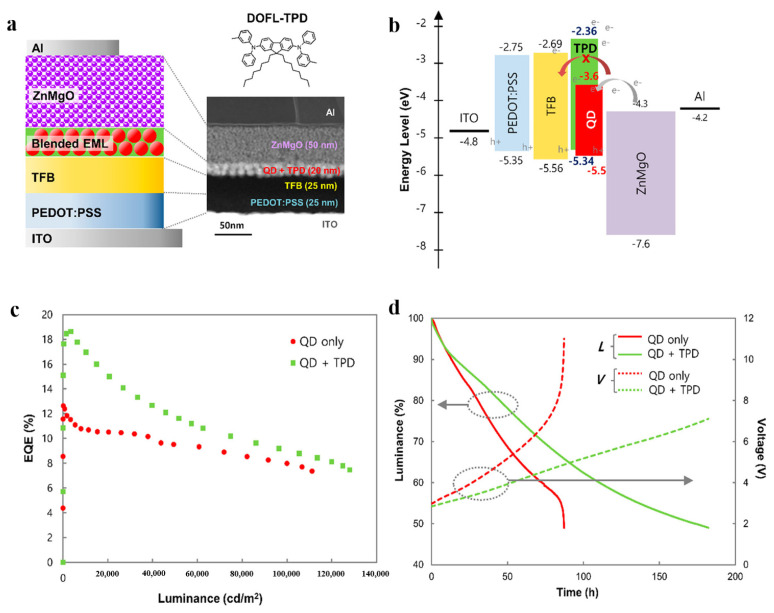
(**a**) Schematic device illustration (left), molecular structure of DOFL−TPD, and cross−sectional TEM image of QLEDs with DOFL−TPD blended QDEML (right). (**b**) Energy diagram of QLED comprising DOFL−TPD blended QDEML. (**c**) EQE−Luminance characteristics for the QLEDs with QD−only EML and DOFL−TPD blended QDEML. (**d**) Operational lifetime of QLEDs with QD−only EML and the blended QDEML measured at initial luminance of 5750 cd/m^2^. Reprinted with permission from Ref. [25]. © 2021, American Chemical Society.

Mixed QDs with conducting polymers as EML is a good strategy to improve the charge injection efficiency to balance the carriers [94]. Han et al. fabricated a uniform thin film by blending the organic molecule N,N’-bis (3-methylphenyl)-N,N’-bis-(phenyl)-9,9-dioctylfluorene (DOFL-TPD) with long-alkyl-chains-covered InP/ZnSe/ZnS QDs, as shown in Figure 12 [25]. They found that DOFL-TPD was uniformly distributed in the blended EML without any phase separation, which facilitated hole injection and energy transfer to the quantum dots; moreover, the blended EML exhibited a highly efficient electron-blocking ability for HTL, thereby enhancing the efficiency and lifetime of QLED devices. The device of the blended EML exhibits an EQE of 18.6%. Compared with pure InP/ZnSe/ZnS QLEDs without DOFL-TPD, the lifetime is significantly prolonged with a lifetime of 107,772 h at a luminance of 100 cd/m^2^. The idea of homogeneously mixing QDs with efficient charge transport materials to prepare the blended EML shows great promise for developing efficient optoelectronic devices.

### 4.2. Modification of ETL

**Figure 13:**
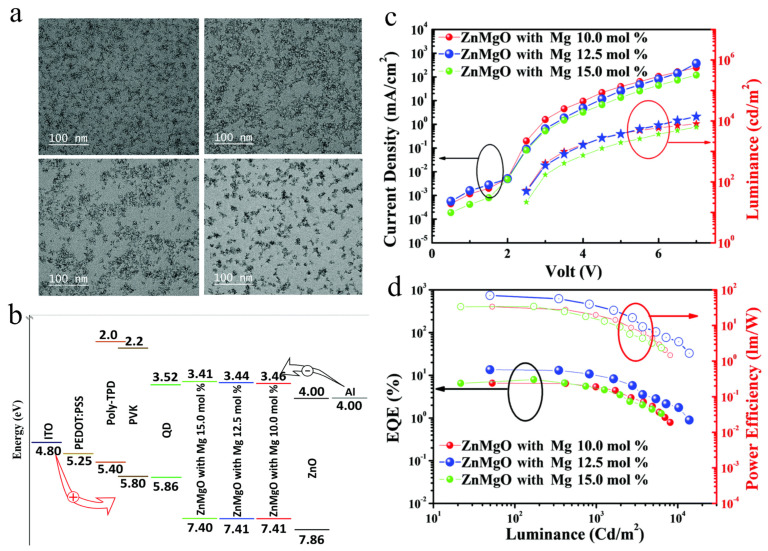
TEM images of (**a**) ZnO. ZnMgO with (**b**) 10.0 mol% Mg, and (**c**) 12.5 mol% Mg, and (**d**) 15.0 mol% Mg. (**b**) Band structure diagram of the InP QLED. (**c**) Current density−voltage−luminance curves of QLEDs fabricated with ETLs with different Mg contents. (**d**) EQE−luminance−power efficiency curves of QLEDs fabricated with ETLs with different Mg contents. Reprinted with permission from Ref. [33]. © 2019, The Royal Society of Chemistry.

In a previous work, ZnO nanoparticles (NPs) [95] and doped-ZnO NPs [96,97] with high mobility, suitable energy level, and solution-process deposition, were identified as the best electron transport material for QLEDs. In addition, the strong electron injection ability of the ZnO NP layer could result in excess electrons in EML and form an undesirable leakage current. In general, the excess electrons may cause many problems, such as charged QDs, reduction of the fraction of excitons, trion emission, poor EL performance, or drop of device lifetime [98]. In 2017, Wang et al. replaced ZnO with ZnMgO as the ETL of InP QLEDs, which greatly improved the device performance, giving it a highest brightness level of over 10,000 cd/m^2^ [82]. Although ZnO has high electron mobility, Wang et al. believes that the electron injection barrier is still large. As an alternative, ZnMgO, with a higher CBM, was used as an ETL in InP QLEDs to reduce the electron injection barrier, thereby improving the performance of InP QLEDs. In 2019, Lee et al. acknowledged that the excess electrons in InP QLEDs are the main reason for the emergency of non-radiative recombination process, and the electron mobility can be reduced by Mg doping; and green InP QLED with 12.5 mol% Mg the ZnMgO ETL reaches a maximum brightness of 13,900 cd/m^2^ and an EQE of 13.6%, as shown in Figure 13 [33]. For another point of view, Wu et al. argued that the improvement of InP QLEDs with ZnMgO ETL is due to the passivation of the band gap states and reduction of the electron conductivity [70]. For QLEDs with lower conductivity ZnO, the exciton quenching at the interface between QD and ZnO is smaller, resulting in a higher EQE and current efficiency.

### 4.3. Modification of HTL

**Figure 14:**
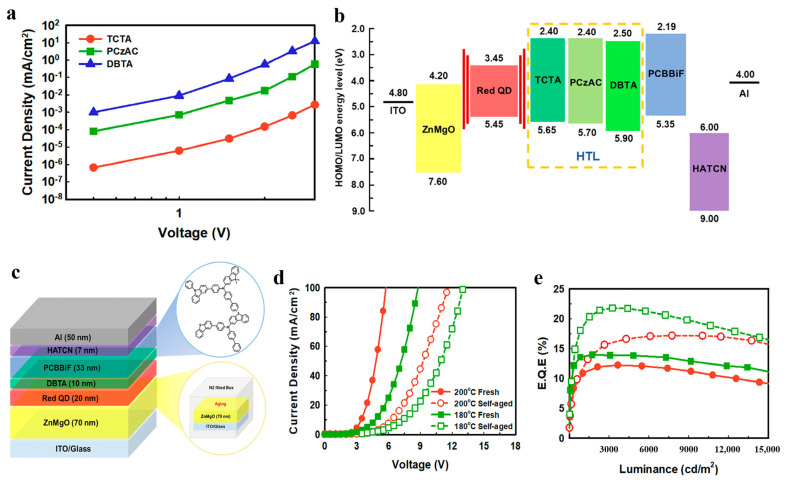
(**a**) Current density−voltage curves for different HTL materials. (**b**) Device structure and HOMO and LUMO energy levels of each part. (**c**) Schematic diagram of the device structure. EL performance: (**d**) current density−voltage and (**e**) EQE−brightness curves of QLEDs fabricated with Zn_0.83_Mg_0.17_O layers by different processes. Reprinted with permission from Ref. [23]. © 2020, American Chemical Society.

Most of the above improvements in device performance were achieved by suppressing the excess electrons. The more desirable approach to circumvent the influence of unbalanced charge injection is to enhance the injection and transport of holes in order to enhance the EL performance. The modification of HTL and hole injection layer (HIL) has also been proved feasible by many works, for example, multilayer HTLs with cascaded energy levels or add dipole layers between HTL and EML to achieve energy level alignment [99,100,101]; and doping hole transport materials to improve the conductivity [102]. Kim et al. used 2,3,4,6-tetrafluoro-7,7,8,8-tetracyanoquinodimethane (F4-TCNQ) as the p-type dopant material and successfully diffused into the middle of HTL and HIL in the form of an interlayer through thermal annealing [103]. This p-type dopant HTL enhances the hole injection efficiency and improves the charge balance of InP QLEDs, and the EQE increased from 1.6% to 3.78%. Yeom et al. believes that the ETL with higher electron mobility and the HTL with low hole mobility cause exciton quenching and charge unbalance [23]. Therefore, a new HTL with high hole mobility and deep highest occupied molecular orbital (HOMO), N-([1,1′-biphenyl]-4-yl)-N-(4-(dibenzo[b,d]-thiophen-2-yl)phenyl)dibenzo[b,d]thiophen-2-amine (DBTA), was designed to transport holes to InP QDs faster and more efficiently. The inverted red InP QLED reaches a current efficiency of 23.4 cd/A, an operating lifetime of 72,848 h at 100 cd/m^2^ and an EQE of 21.8%, which is the highest EQE value for red InP QLEDs, as shown in Figure 14. Zhu et al. conducted a systematic study on the simultaneous optimization of ETL and HTL [104]. First, double HTLs ((poly [(9,9-dioctylfluorenyl-2,7-diyl)-co-(4,4′-(N-(p-butylphenyl))-diphenylamine)], poly (9-vinlycarbazole), TFB/PVK) was used to form a step-shaped implantation, which greatly improved the film morphology at the interface between HTL and QDs. Compared with the device using only PVK, the turn-on voltage was reduced from 2.8 to 2.6 V, and the current efficiency rose from 3.16 to 4.13 cd/A. The PVK layer was further doped with TAPC to enhance the hole injection efficiency; finally, a peak current efficiency of 7.58 cd/A was obtained, which is higher than that of the PVK-only device.

At present, the selection of HTL for InP QLEDs mainly focuses on overcoming the large hole injection barrier. However, some researchers put forward different ideas; they acknowledged that the lowest unoccupied molecular orbital (LUMO) of HTL has an important influence on the EL efficiency [70,105]. Compared with red InP QLEDs, green InP QLEDs have more obvious parasitic emission problems due to the smaller size of InP QDs. Kim et al. used ITO/PEDOT:PSS/TFB (or PVK)/InP QDs/ZnO/Al structures to compare the effect of TFB and PVK as HTL [105]. The researchers used large-scale red InP QDs with strong electron confinement and, therefore, less influence from parasitic emission as the light-emitting layer and observed that QLEDs with PVK as HTL almost completely suppressed the parasitic emission, while QLEDs with TFB as HTL could still have a small number of parasitic emissions. The reason is that the LUMO energy level of PVK is 0.4 eV higher than that of TFB, and the higher LUMO of PVK sets a higher potential barrier for electrons in InP QDs than using TFB, thus limiting the leakage of electrons and suppressing the generation of parasitic emission. Wu et al. replaced TFB with poly [bis(4-phenyl) (2,4,6-trimethylphenyl)amine] (PTAA), which has a higher LUMO and the highest occupied molecular orbital (HOMO) energy levels compared to HTL, and observed that the parasitic emission of HTL basically disappeared [70]. This is consistent with the idea that the higher LUMO of HTL prevents electron injection in the quantum dots and, thus, suppresses parasitic radiation.

### 4.4. Modification of Electrode and Top-Emitting InP QLED

**Figure 15:**
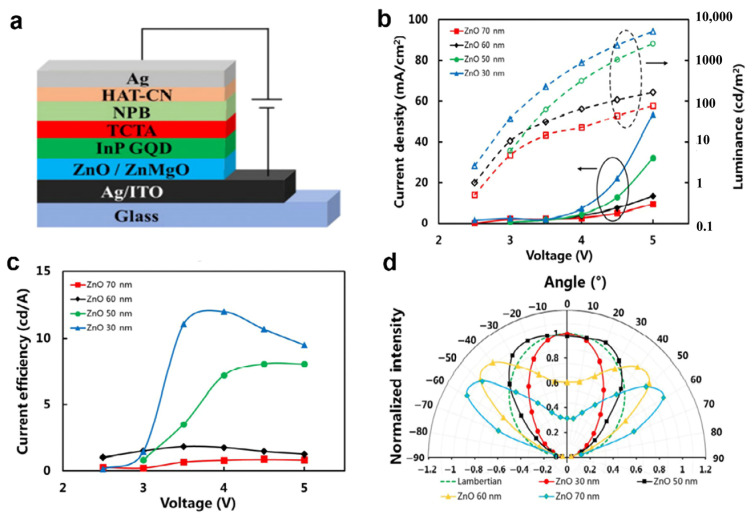
(**a**) Basic device structure of InP QLED. Performance of the top-emission QLEDs: (**b**) J−V−L characteristics and (**c**) current efficiency curves with different ZnO ETL thickness. (**d**) The measured normalized angle−dependent EL intensity of the top-emission QLEDs with different ZnO ETL (the HTL thickness is 30 nm). Reprinted with permission from Ref. [110]. © 2021, Tsinghua University Press and Springer−Verlag GmbH Germany, part of Springer Nature.

To further enhance the luminance efficiency of QLEDs, an electrode with high transmittance film or optimization of the angular distribution by top-emitting (TE) device structure is deposited in the InP QLED. In 2016, to realize the transparent InP QLED display, Kim et al. proposed a two-step sputtering process of indium zinc oxide (IZO) top electrode to green InP QLED, and the transparent QLED reached a transmittance of more than 74% for the whole device array [106]. In 2017, Kim et al. used smooth, flexible, and transparent Ag nanowires (AgNWs) as the bottom electrode to fabricate a green InP QLED. The flexible InP QLED gave a durable performance even under bending with a curvature radius of 5 mm [107]. In 2019, Lee et al. reported inverted TE InP QLED by introducing a hole-suppressing interlayer [108]. The green-emitting InP QLEDs reached a maximum current efficiency of 15.1–21.6 cd/A and a maximum luminance of 17,400–38,800 cd/m^2^, and the green and red TE InP QLEDs exhibited a FWHM of 37 and 38 nm, respectively. In 2020, Park rt al. demonstrated efficient and environmentally friendly TE InP QLEDs, which were achieved by employing top-emitting structure. Consequently, the optical simulation was satisfied, and the extraction of QD emission was considerably enhanced, realizing a 3.2-fold improvement compared to those of the bottom emission device [109]. In 2021, Li et al. used a reflective Ag/ITO (Figure 15) as the bottom electrode and investigated a TE green InP QLED [110]. By optimizing the angular distribution, the devices exhibit a maximum current efficiency of 30.1 cd/A and a narrowed FWHM of 31 nm, which is a record value for green InP QLED to date.

### 4.5. Optical Modification

**Figure 16:**
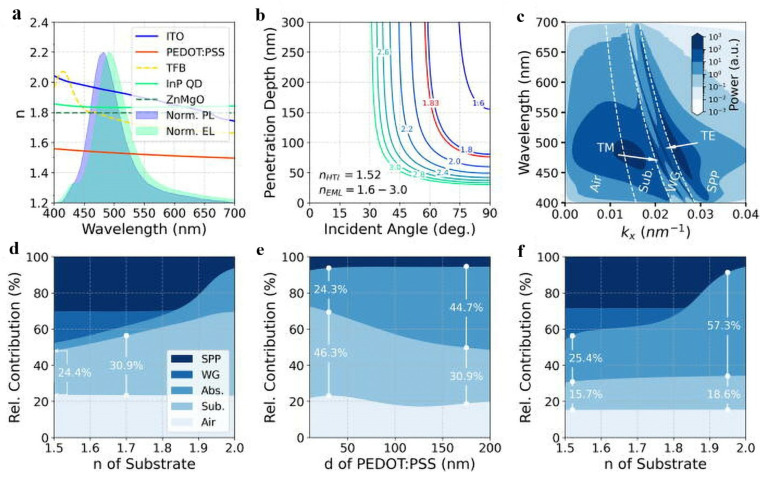
(**a**) The refractive indices of the QLED materials, the normalized PL spectrum of the InP QD, and the EL spectrum of the InP QLED. (**b**) Penetration depth of the evanescent wave vs. angle of incidence for varied refractive index of EML at wavelength of 488 nm. (**c**) Power dissipation spectrum of the InP QLED; dashed lines separate the regions of different optical modes. Optical power distribution of different optical modes (**d**) vs. refractive index of the substrate at PEDOT:PSS thickness of 30 nm, (**e**) vs. PEDOT:PSS thickness at refractive index of the substrate of 2, and (**f**) vs. refractive index of the substrate at PEDOT:PSS thickness of 600 nm. Reprinted with permission from Ref. [26]. © AIP Publishing.

The device efficiency of QLEDs is not only affected by the device material and energy level structure, but also by the light outcoupling efficiency (LOE) of the device [111,112]. The LOE is mainly determined by the device structure. In the multilayer structure of QLEDs, the charge transport layer adopts the form of organic–inorganic composite, and the HTL composed of organic materials usually has a lower refractive index than that of inorganic materials. Moreover, the refractive index of the device material is much larger than that of air [112]. The photons are consumed in the waveguide mode and the plasmonic mode, and this affects the light outcoupling of the QLED and reduces the device efficiency. Generally, LOE in a conventional planar LED architecture can be improved by modifying the shape or the surface of the devices, using an outcoupling lens, or introducing a microcavity [26,113,114,115]. To extract light from waveguide modes to air modes, in 2022, Mei et al. proposed a light extraction strategy by using a thin HTL, a high-index substrate, and substrate surface-roughening (Figure 16) to enhance the EQE of bule InP QLED [26]. As a result, light extraction efficiency has been significantly improved, leading to an EQE of 2.8%, which is a record value for blue InP QLED to date.

## 5. Challenge and Future Direction

InP QLEDs show great potential in the field of lighting display, due to their environmental friendliness, low toxicity, and excellent optoelectronic properties. The currently reported maximum EQEs of RGB InP QLEDs are 21.8%, 16.3%, and 2.8% [23,24,26], respectively. Though the EL performance of red QLED can be comparable to that of Cd-based QLEDs, the EL characteristics of green and blue QLEDs are still far from those of Cd-based QLEDs. There are still many challenges to achieving an environmentally friendly wide color gamut display.

### 5.1. High-Efficiency Green and Blue InP QLEDs

How to obtain high-efficiency and narrow-linewidth green and blue InP QLEDs is the key factor to realize commercial application. Green and blue InP QLEDs still have the following key scientific issues to be solved; that is, the alloy structure and size distribution control of InP QDs must be considered, especially in regard to how to use multi-shell layers to suppress interface strain and trap defects. For green and blue InP QDs, a smaller size of core is required for synthesis [116], which easily generates more surface and interface defects and suppresses the formation of a perfect epitaxial shell. How to choose a suitable material, especially a suitable intermediate shell material, is something that needs to be seriously considered. In the optimization of the device structure, it is necessary to select a suitable charge transport material, adjust its energy level through doping or surface modification, and combine the optical optimization of the device to improve the light out-coupling efficiency and charge injection efficiency and greatly improve the EQE and brightness of QLED.

### 5.2. Narrow Linewidth InP QLEDs

A thick shell or alloyed shell is usually used to passivate the surface traps at the InP/ZnS interface that are caused by oxidation defects or component doping [20,66,67]. However, the lattice mismatch of the core/shell structure implies more defects and prevents the formation of a perfect heteroepitaxial interface. In addition, broad emission originates from the luminescence of holes trapped by surface and lattice defects, resulting in spectral broadening [28,117]. For narrow linewidth InP QLED, the growth of InP QDs with high lattice integrity, uniformity, and high spherical symmetry is helpful to exhibit more excellent light-emitting properties. By increasing the size of QDs and the thickness of the shell and passivating the surface to reduce trap sites, we can increase the ability to inhibit nonradiative recombination such as AR and FRET. In addition, optical structure optimization is also an effective means to obtain a narrow linewidth. The planar optical microcavity can improve the light output efficiency of the QLEDs, adjust the mode density, and realize the narrowing of the spectrum, which is an efficient strategy for spectral narrowing.

### 5.3. Planar Microcavity InP QLEDs

It is demonstrated that the optical optimization of the QLEDs is as important as the electrical optimizations that were mentioned above. The incorporation of optical microcavity structures into QLEDs is a very effective strategy to enhance the EL performance of the device by simultaneously enhancing the QLED spectral intensity, narrowing the spectral width, and improving the efficiency. A microcavity is a structure with at least one dimension on the order of an optical wavelength [118,119]. Fabry–Perot microcavity can be easily constructed in planar-structured QLEDs that consist of a QD emitting layer placed at the antinode of the cavity. Consequently, improved LOE can be realized by the coupling of light emission into the optical modes of the cavity. Wang et al. fabricated a set of red, green, and blue QLEDs with high EQE by incorporating microcavity structures with a distributed Bragg reflector (DBR) and Al as reflectors [120]. Bragg mirrors, in fact, are one-dimensional (1D) photonic crystals (PhCs) which reflect light due to the photon confinement band effect [121]. The photon confinement band of a 1D PhC depends on the angle of incidence, so Bragg mirrors do not completely solve the problem of light reflection. In 2002, Ferrini et al. presented first internal light source (ILS) transmission measurements on 2D PhC etched in GaInAsP/InP slab waveguide structures [122]. Obviously, 3D PhCs with complete forbidden bands are even more ideal light reflectors [123,124,125]. The fabrication and optical characterization of an InP-based L3 PhC microcavity embedded with moderate-density traditional epitaxial InAs/InP QDs emitting at telecommunication wavelengths was reported in 2017 [126]. QDs emit highly linearly polarized light at telecommunication wavelengths with spectral linewidths below 50 μeV. In 2021, Bian et al. performed a comparative simulation study of void-containing and all-semiconductor PhC surface-emitting lasers with square lattices and round atoms [127]. They demonstrate that the void-containing structure can achieve a higher coupling coefficient than the all-semiconductor structure. The mentioned reports of conventional epitaxial InP-based PhC devices shows a promising approach to obtain high-efficient colloidal InP QLEDs.

### 5.4. Flexible InP QLEDs

With the development of electronic science and technology, flexibility and wearability have gradually become two of the important trends in the future development of optoelectronic devices [128,129]. While the flexible Cd-based QLED luminescence performance has made great progress in recent years [130,131,132], the progress of flexible InP QLED is relatively slow. The research and development of flexible QLED is of great significance to the industrialization process of QLED. Compared with flexible OLEDs, solution-processed flexible InP QLEDs have more stringent requirements on substrates and transparent electrodes, because the device performance of QLEDs is more sensitive to fluctuations in the thickness of each functional layer. The transparent electrodes used in the reported flexible Cd-based QLEDs mainly include ITO and translucent thin metals [133]. However, due to the high brittleness of ITO, it is prone to cracks after repeated bending, resulting in a sharp increase in sheet resistance, which seriously affects the EL performance. At the same time, indium, the main raw material of ITO, is extremely rare, and that makes the preparation cost of ITO increase year by year. The advantage of translucent thin metal is the easy fabrication, but its low transmittance will seriously limit the EL performance of the device. In recent years, Ag nanowire has been found to be an excellent flexible transparent electrode which can achieve high electrical conductivity and transmittance [107]. At the same time, dielectric–metal–dielectric multilayer transparent electrodes have been widely used in various optoelectronic devices and achieved good device performance, especially their excellent bending resistance, and large-area uniformity characteristics, which make it show important potential in the application of constructing flexible optoelectronic devices.

## Figures and Tables

**Table 1:** Electroluminescent (EL) performance of RGB InP QLEDs.

QD Structure	EL (nm)	FWHM (nm)	EQE (%)	Luminance (cd/m^2^)	Reference
InP/ZnSe/ZnS	630	35	21.4%	100,000	[6]
InP/ZnSe/ZnS	630	34	18.6%	128,577	[25]
InP/ZnSe/ZnS	632	36	21.8%	23,300	[23]
InP/ZnSe/ZnS	545	39	16.3%	12,600	[24]
InP/ZnSe/ZnS	531	34	13.6%	13,900	[33]
InP/ZnS	492	/	2.8%	421	[26]

**Table 2:** Representative synthesis methods of InP QDs and EQE of QLED.

QD Structure	Methods	EL (nm)	PLQY (%)	FWHM (nm)	EQE (%)	Reference
InP/ZnSe/ZnS	Hot-injection	630	96	34	18.6	[25]
InP/ZnSeS/ZnS	Hot-injection	525	95	45	7.06	[47]
In(Zn)P/ZnSe/ZnS	Heat-up	641	67	36	/	[19]
InP/ZnS/ZnS	Heat-up	484	43		1.47	[48]
InP/ZnSe/ZnS	Seeded growth	533	65 ± 2	37		[41]
InP/ZnSe/ZnS	Seeded growth	630	100	35	21.4	[6]
InGaP/ZnSeS/ZnS	Cation exchange	465	80	45–47	2.5	[43]
Cu-assisted InP/ZnS	Cation exchange	~420	25	~72	/	[49]
